# Exploring patient’s clinical outcomes, hospital costs, and satisfaction after the implementation of integrated clinical pathway-based nursing practice model

**DOI:** 10.1186/s12962-025-00645-5

**Published:** 2025-07-30

**Authors:** Rini Rachmawaty, Elly Wahyudin, Agussalim Bukhari

**Affiliations:** 1 Department of Nursing Management, Faculty of Nursing, Hasanuddin University, Makassar, Indonesia; 2 Department of Clinical Pharmacy, Faculty of Pharmacy, Hasanuddin University, Makassar, Indonesia; 3 Department of Nutritional Sciences, Faculty of Medicine, Hasanuddin University, Makassar, Indonesia

**Keywords:** Clinical outcomes, Hospital expenses, Integrated clinical pathway, Length of stay, Nursing care quality, Patient satisfaction

## Abstract

**Introduction:**

Hospitals provide essential health services, focusing on quality, safety, and patient-centered care. The rising prevalence of communicable and non-communicable diseases in Indonesia has led to increased National Health Insurance (NHI) costs, with communicable diseases representing a significant burden despite global progress in disease control. Tuberculosis remains a major global health concern, causing 1.25 million deaths in 2023, while Indonesia ranks second globally for tuberculosis burden, accounting for 10% of global TB cases with an estimated 969,000 cases annually. Despite efforts to improve quality, Haji and Labuang Baji Hospitals face challenges, including financial losses and suboptimal patient outcomes. This study aims to assess the impact of an Integrated Clinical Pathway (ICP)-based Professional Nursing Practice Model on clinical outcomes, hospital costs, and patient satisfaction at these hospitals.

**Methods:**

This study employed action research methodology by developing, implementing, and evaluating the clinical guidelines and ICP for 10 diseases that were categorized as high volume, high risk, and high cost based on secondary data analysis using the NHI databases. Data was collected through observation of the ICP implementation on 40 patients and interviews of Professional Care Providers (PCPs) and was analyzed using IBM SPSS Statistics version 28.

**Results:**

A total of 40 patients from Haji Hospital (*n* = 20) and from Labuang Baji Hospital (*n* = 20) with diagnoses of pulmonary tuberculosis, pneumonia, dyspepsia, typhoid fever, normal delivery, and COPD were included and analyzed. Compliance with ICPs in both hospitals was quite low: 50.02% in Haji Hospital and 44.46% in Labuang Baji Hospital. However, the length of patients’ stays (LOS) generally complied with ICP standards, although some cases exceeded the benchmarks. Hospital costs varied across different disease diagnoses, impacting overall financial outcomes. Patient satisfaction improved across all diagnostic categories.

**Conclusion:**

The implementation of ICP at both hospitals showed that all indicators of patient clinical outcomes improved according to the time specified in ICP, resulting in patients’ LOS being shorter than stipulated in ICP and an increase in patient satisfaction. ICP implementation has also led to hospitals earning different profits in almost all diagnoses.

## Background

Hospitals are health service institutions that provide comprehensive individual health services including inpatient, outpatient, and emergency services. Quality health services are characterized by safety, timeliness, efficiency, effectiveness, patient-orientation, fairness, and integration [1]. According to the Regulation of the Minister of Health of the Republic of Indonesia, hospitals must provide reports on the fulfilment of national indicators of health service quality and patient safety incidents to the Ministry of Health [2].

Communicable diseases (CDs) and non-communicable diseases (NCDs) remain significant global health concerns. Globally, communicable diseases continue to pose substantial challenges despite advances in prevention and treatment. Tuberculosis remains a major global health threat, causing 1.25 million deaths worldwide in 2023 [3]. Lower respiratory infections, primarily pneumonia, are among the leading causes of death globally, responsible for approximately 2.4 million deaths annually [4].

In Indonesia, both CDs and NCDs represent critical health challenges. Indonesia ranks second globally for tuberculosis burden, accounting for approximately 10% of global TB cases [5]. The country also faces significant challenges with other communicable diseases that are commonly treated in hospital settings. Pneumonia remains a leading cause of hospitalization and mortality, particularly among vulnerable populations including children and the elderly [6]. Typhoid fever, an endemic disease in Indonesia, continues to present substantial healthcare burdens, requiring standardized care pathways to ensure optimal treatment outcomes. Indonesia is classified among countries with high or very high typhoid fever incidence according to recent Global Burden of Disease estimates, with environmental factors such as poor sanitation and crowding contributing to sustained transmission across the archipelago [7].

The dual burden of communicable and non-communicable diseases has led to an annual increase in National Health Insurance (NHI) claim costs by 18–25%, totaling approximately IDR 118.16 trillion (approx. USD 7.13 billion) between 2014 and 2020 [8]. At Haji and Labuang Baji Hospitals, both CDs and NCDs are among the top ten diseases characterized by high volume, risk, and cost, necessitating efficiency measures without compromising service quality [9]. Despite governmental efforts, optimal health service quality has yet to be achieved.

To enable year-to-year comparison and assess emerging efficiency trends in disease-related hospitalizations, data from early 2020 were included despite the onset of the COVID-19 pandemic, as both Haji and Labuang Baji Hospitals continued to manage substantial caseloads during this period [9]. At Haji Hospital, NHI patient casemix data from January 1 to December 31, 2019, reported 8,778 total admissions, with 10.9% involving readmissions, 3.5% with hospital stays exceeding ten days, and 21.1% resulting in financial losses up to IDR 103,353,082 (approx. USD 6,237.5) [9]. From January 1 to March 30, 2020, the hospital admitted 2,794 patients, of whom 4.6% were readmitted, 3.4% experienced prolonged stays, and 19.9% generated financial losses amounting to IDR 316,167,489 (approx. USD 19,076.9) [9]. Although hospital operations may have been affected by the onset of the COVID-19 pandemic, these data remain relevant for observing trends in disease admissions, readmission rates, length of stay, and financial losses. This comparison helps to assess the consistency of hospital performance and cost-efficiency efforts prior to and during the early pandemic period [9].

A comparable pattern emerged at Labuang Baji Hospital. In 2019, among 5,051 total admissions, 12.7% were readmissions, 9.4% exceeded ten days of hospitalization, and 30.3% led to financial losses up to IDR 93,251,164 (approx. USD 5,626.5) [9]. During the subsequent period from January 1 to July 31, 2020, 2,675 patients were admitted, with 8.3% readmitted, 8.9% staying over ten days, and 27.5% incurring losses reaching IDR 38,461,669 (approx. USD 2,320.7) [9]. Notably, negative cost margins were observed even among patients hospitalized for only 1–3 days, underscoring systemic inefficiencies beyond length of stays (LOS)-related expenditures [9]. These findings emphasize the persistent financial strain posed by disease admissions and validate the need for improved care pathways and resource optimization.

According to the Regulation of the Minister of Health of the Republic of Indonesia Number 30 of 2022, compliance with clinical pathways and patient satisfaction are part of the quality indicators at hospitals [10]. In 2018, the government paid more attention to hospital claims due to the NHI fund deficit and encouraged the use of clinical pathways to provide medical services and review medical classifications [11]. Clinical pathways become the recommended objective tool to improve the quality and safety of patient care outcomes for hospitals to comply with and utilize health reimbursement systems. Well-designed clinical pathways can support healthcare teams to deliver key management priorities in a timely manner [12]. Additionally, patient satisfaction is another crucial hospital quality indicator. The patient satisfaction rate ranges from 78.86 to 84.73%, which should be ≥ 90% in 2020 at Haji Hospital [13]. Patient satisfaction with healthcare quality was also found to vary between inpatient classes, with patients treated in grade 3 having the lowest level of satisfaction [14].

This study aims to evaluate clinical outcomes, hospital costs, and patient satisfaction following the development of an Integrated Clinical Pathway (ICP)-based Professional Nursing Practice Model at Haji and Labuang Baji Hospitals in Makassar. As two of seven regional public hospitals governed by the South Sulawesi Provincial Government, these facilities deliver essential secondary and tertiary care under Indonesia’s NHI scheme. Their strategic role and public ownership make them priority sites for quality improvement initiatives. Assessing ICP implementation in these settings offers valuable insights into standardized care practices in regional public hospitals. The evaluation framework comprised three key outcome domains: (1) clinical outcomes to assess treatment effectiveness, (2) hospital cost profiles to determine financial efficiency, and (3) patient satisfaction to evaluate the quality and responsiveness of care. These indicators collectively reflect the intended goals of ICP implementation.

## Methods

### Study design and setting

This study employed a quantitative descriptive design to evaluate the implementation of ICPs for ten high-burden conditions in two provincial hospitals. The ten conditions comprise pulmonary tuberculosis (TB), pneumonia, chronic obstructive pulmonary disease, bronchopneumonia, dyspepsia, typhoid fever, normal delivery, diabetic ulcer, chronic kidney disease stage 5, and breast cancer [5]. The evaluation encompassed three domains: (1) the implementation of ICPs in clinical practice, (2) the financial efficiency of care delivery, and (3) patient satisfaction with the services received.

The study was conducted from February 9 to April 9, 2023, in two public referral hospitals located in South Sulawesi Province, Indonesia: Haji Hospital and Labuang Baji Hospital. Both institutions are managed by the South Sulawesi Provincial Government and were purposively selected based on their (a) status as provincial tertiary care hospitals, (b) high patient volumes, (c) active participation in the NHI scheme, and (d) prior implementation of ICPs for several priority diseases.

### Operational definition of ICP

An ICP is defined in this study as a structured, multidisciplinary care plan used to guide evidence-based management of specific diagnoses throughout a patient’s hospitalization. Each ICP includes standardized procedures for clinical assessment, diagnostics, interventions, documentation, and discharge planning. The ICPs evaluated were officially adopted by the participating hospitals and designed to ensure quality, consistency, and efficiency in patient care [15].

### Sampling and participants

A purposive sampling strategy was used to recruit patients managed under ICPs during the study period. Each hospital contributed 20 participants, resulting in a total of 40 patients. This sample size was determined based on practical considerations related to real-world hospital settings and the exploratory nature of the research. Although conventional power analysis recommends at least 26 participants per group to detect large effect sizes (d = 0.80) with 80% power at a 5% significance level [16], previous research has demonstrated that smaller sample sizes (e.g., 12–20 per group) can yield meaningful results when clinical outcomes are well-defined, patient populations are relatively homogeneous, and interventions are embedded within routine care [17, 18]. Therefore, a sample size of 20 per group was considered sufficient to detect moderately large effects in this comparative observational study and to generate preliminary data for future large-scale investigations.

#### Inclusion criteria

Participants were included if they met the following criteria:

- Adult patients aged ≥ 17 years. Although international standards typically define adulthood as ≥ 18 years, this study included participants aged 17 and above in alignment with Indonesian civil law, which recognizes individuals at this age as eligible for a national identity card and capable of providing informed consent for research participation [19].
- Diagnosed with one of the ten medical conditions included in the ICPs under evaluation: pulmonary TB, pneumonia, chronic obstructive pulmonary disease (COPD), bronchopneumonia, dyspepsia, typhoid fever, normal delivery, diabetic ulcer, chronic kidney disease stage 5, or breast cancer.
- Admitted to the inpatient unit via the emergency department or outpatient clinic.
- Covered by health insurance.

#### Exclusion criteria

Patients were excluded from the study if they:

- Requested discharge against medical advice during treatment,
- Died during the hospitalization period, or.
- Had clinical complications present at the time of initial diagnosis.

### Data collection

Data collection was conducted by trained researchers through systematic observation and targeted interviews over a two-month period (February 9 to April 9, 2023). The study employed three integrated assessment components:

1. **Integrated Clinical Pathway (ICP) Implementation Assessment**: Direct observation of clinical care delivery was conducted using a standardized ICP checklist comprising 14 core components: initial medical and nursing assessments; diagnostic evaluations (laboratory and radiological); specialist consultations; follow-up assessments (medical, nursing, nutritional, and pharmaceutical); formulation of diagnoses across disciplines; discharge planning; interdisciplinary patient education; medical treatment administration; therapeutic interventions; ongoing monitoring and evaluation; mobilization and rehabilitation; anticipated clinical outcomes; clearly defined discharge criteria; and structured education to support continuity of care post-discharge [15]. Each component was scored as compliant (1) or non-compliant (0) based on adherence to established ICP protocols and adequate documentation in patient records, with overall compliance calculated as (completed items/total items) × 100%.
2. **Financial Efficiency Assessment**: Hospital cost profiles were analyzed through examination of patient care processes, including direct medical costs (medications, diagnostics, procedures), accommodation costs, and professional fees. Cost data was sourced from the hospital’s financial department and patient medical records [15].
3. **Patient Satisfaction Measurement**: Patient satisfaction was assessed using a validated questionnaire adapted from a previous study [14]. The instrument evaluated satisfaction across multiple domains, including healthcare provider interactions, facility quality, and overall care experience. Patient satisfaction scores were categorized into three levels—very satisfied, satisfied, and quite satisfied—based on predefined score ranges established through quartile analysis of the satisfaction score distribution.

### Qualitative component

Individual interviews with Professional Care Providers (PCPs) were conducted during the observation period using open-ended questions to explore barriers and facilitators to ICP implementation. These interviews were strategically conducted when clarification on service delivery processes was needed during the observation period, thereby providing deeper insights into implementation challenges and contextual factors affecting compliance rates. This approach allowed researchers to gain contextual understanding of implementation processes and obtain real-time explanations of observed practices, enhancing the comprehensiveness of the assessment.

### Instrumentation

Three validated instruments were employed for data collection. The ICP compliance checklist was developed based on established clinical guidelines and underwent content validity review by clinical experts to ensure appropriateness for the hospital settings. The financial efficiency assessment tool was used to systematically collect hospital cost data from financial departments and patient medical records. The patient satisfaction questionnaire was adapted from a previously validated instrument [14] with demonstrated reliability in similar hospital environments. Internal consistency reliability was confirmed through Cronbach’s alpha analysis (α = 0.767) [14, 15].

### Minimizing the hawthorne effect

Given that this study employed direct observation to assess ICP implementation by physicians, nurses, nutritionists, and clinical pharmacists, the potential for the Hawthorne effect was carefully considered. To reduce this bias, research assistants were thoroughly trained to observe in a discreet, consistent, and non-disruptive manner. Prior to data collection, they were introduced to healthcare teams to promote familiarity and ease initial reactivity. Observations were conducted over a continuous two-month period, from February 9 to April 9, 2023. This extended timeframe allowed clinical staff to acclimate to the presence of observers, thereby promoting natural behavior and minimizing observation-induced changes in practice.

### Data analysis

All quantitative data were analyzed using IBM SPSS Statistics version 28. Descriptive statistics, including frequencies (n), percentages (%), means, standard deviations (SD), and ranges (minimum-maximum values), were calculated to summarize patient characteristics and study variables.

Comparative analyses between the two hospitals were conducted to assess baseline patient characteristics and identify potential confounding factors that might influence outcome interpretation. *Chi-square* tests were applied for categorical variables, while independent *t*-tests were used for continuous variables. Statistical significance was set at *p* < 0.05.

ICP compliance rates were categorized using predefined thresholds: high compliance (> 75%), moderate compliance (50–75%), and low compliance (< 50%). Patient satisfaction levels were classified into three categories—very satisfied, satisfied, and quite satisfied—based on predefined score ranges derived from the validated questionnaire scoring system.

## Results

### Patient characteristics

A total of 40 patients from Haji Hospital (*n* = 20) and Labuang Baji Hospital (*n* = 20) with diagnoses of pulmonary tuberculosis, pneumonia, dyspepsia, typhoid fever, normal delivery, and COPD were included and analyzed. While ICPs were developed for 10 diseases, only these 6 diagnoses had sufficient patient admissions during the study period to be included in the analysis.

Table 1 shows the average age of patients at Haji Hospital was 51.15 years (range 29–70 years), while the average age at Labuang Baji Hospital was 44.35 years (range 17–73 years). 45% of patients from both hospitals graduated from high school. Many patients were housewives: 35% at Haji Hospital and 45% at Labuang Baji Hospital. Most patients were diagnosed with pulmonary TB: 30% at Haji Hospital and 55% at Labuang Baji Hospital.

**Table 1:** Characteristics of respondents at Haji hospital (*n* = 20) and Labuang Baji hospital (*n* = 20)

Characteristic	Haji Hospital (*n* = 20)		Labuang Baji Hospital (*n* = 20)		*p*
	M (± SD)	Min-Max	M (± SD)	Min-Max	
**Age (Years)**	51.15 (± 12.75)	29–70	44.35 (± 18.52)	17–73	0.093
**Length of Stay (Day)**	4.15 (± 2.11)	1–9	6.20 (± 2.82)	3–12	0.007*
	**n**	**%**	**n**	**%**	
**Sex**					
Male	13	65	7	35	0.056
Female	7	35	13	65	
**Education**					
Not attending school	1	5	0	0	0.141
Elementary school	7	35	3	15	
Junior high school	1	5	4	20	
Senior high school	9	45	9	45	
College	2	10	4	20	
**Occupation**					
Self employed	4	20	2	10	0.021*
Labor/Farmer/Fisherman	5	25	2	10	
Housewife	7	35	9	45	
Student	0	0	3	15	
Other	4	20	4	20	
**Primary Medical Diagnosis**					
Pulmonary TB	6	30	11	55	< 0.001*
Pneumonia	4	20	3	15	
Dyspepsia	6	30	1	5	
Typhoid fever	2	10	4	20	
COPD	0	0	1	5	
Normal Delivery	2	10	0	0	

*Note: M* = Mean; *SD* = Standard Deviation; *n* = Frequency; *%*= Percentage; *Min* = Minimum; *Max* = Maximum;

*p < 0.05 (*Chi-square* test for categorical variables, independent *t*-test for continuous variables); TB = Tuberculosis; COPD = Chronic Obstructive Pulmonary Disease

### Compliance with ICP implementation

Table 2 demonstrates that overall compliance with ICP implementation by PCPs at both hospitals was categorized as low according to predefined criteria. At Haji Hospital, the mean adherence rate was 55.02%, which falls within the moderate compliance range. Among the five diagnoses evaluated, normal delivery achieved the highest compliance at 75.2% (approaching the high compliance threshold), while pneumonia recorded the lowest compliance at 43.1%.

**Table 2:** Compliance with ICP implementation at Haji hospital and Labuang Baji hospital (*n* = 40)

No	Mandatory items in ICP	PCP	Haji Hospital (*n* = 20)										Labuang Baji Hospital (*n* = 20)									
			Pulmonary TB (*n* = 6)		Pneumonia (*n* = 4)		Dyspepsia (*n* = 6)		Typhoid fever (*n* = 2)		Normal Delivery (*n* = 2)		Pulmonary TB (11)		Pneumonia (*n* = 3)		Dyspepsia (*n* = 1)		Typhoid fever (*n* = 4)		COPD (*n* = 1)	
			ICP		ICP		ICP		ICP		ICP		ICP		ICP		ICP		ICP		ICP	
			C (%)	NC (%)	C (%)	NC (%)	C (%)	NC (%)	C (%)	NC (%)	C (%)	NC (%)	C (%)	NC (%)	C (%)	NC (%)	C (%)	NC (%)	C (%)	NC (%)	C (%)	NC (%)
1	Initial Assessment	Physician	50	50	75	25	77.8	22.2	50	50	100	0	54.5	45.5	50	50	66.7	33.3	50	50	50	50
		Nurse	86.1	13.9	100	0	38.9	61.1	100	0	-	-	83.3	16.7	100	0	33.3	66.7	100	0	100	0
		Midwife	-	-	-	-	-	-	-	-	100	0	-	-	-	-	-	-	-	-	-	-
2	Laboratory/Radiology Examination		86	86	14	47.2	52.8	36	64	25	75	20	80	100	0	66.7	33.3	16.7	83.3	30	70	100
3	Consultation	Physician	100	0	75	25	100	0	0	100	0	100	81.8	18.2	0	100	0	100	0	100	0	100
4	Advanced Assessment	Physician	100	0	62.5	37.5	77.8	22.2	100	0	100	0	100	0	100	0	66.7	33.3	100	0	100	0
		Nurse	100	0	62.5	37.5	100	0	100	0	-	-	100	0	50	50	100	0	100	0	66.7	33.3
		Dietitian	16.7	83.3	25	75	5.6	94.4	100	0	-	-	45.5	54.5	0	100	100	0	100	0	100	0
		Pharmacist	0	100	0	100	8.3	91.7	50	50	-	-	0	100	0	100	0	100	0	100	0	100
		Midwife	-	-	-	-	-	-	-	-	100	0	-	-	-	-	-	-	-	-	-	-
5	Diagnosis	Physician	100	0	100	0	100	0	100	0	100	0	100	0	100	0	100	0	100	0	100	0
		Clinical Nutritionist	10	90	-	-	0	100	100	0	-	-	0	100	-	-	0	100	0	100	0	100
		Nurse	22.2	77.8	18.2	81.8	14.3	85.7	22.2	77.8	-	-	22.2	77.8	30.3	69.7	14.3	85.7	19.4	80.6	33.3	66.7
		Dietitian	38.9	61.1	75	25	4.2	95.8	87.5	12.5	100	0	9.1	90.9	0	100	50	50	0	100	0	100
		Midwife	-	-	-	-	-	-	-	-	100	0	-	-	-	-	-	-	-	-	-	-
6	Discharge Planning	Physician	44.4	55.6	-	-	-	-	-	-	-	-	48.5	51.5	-	-	-	-	-	-	0	100
		Nurse	52.4	47.6	25	75	56.7	43.3	0	100	-	-	51.9	48.1	57.1	42.9	20	80	100	0	0	100
		Pharmacist	0	100	0	100	-	-	0	100	-	-	0	100	0	100	-	-	0	100	0	100
		Midwife	-	-	-	-	-	-	-	-	66.7	33.3	-	-	-	-	-	-	-	-	-	-
7	Integrated Education	Physician	50	50	45	55	16.7	83.3	100	0	100	0	40.3	59.7	40	60	50	50	50	50	0	100
		Nurse	59.3	40.7	13.5	86.5	45.2	54.8	44.4	55.6	-	-	42.4	57.6	38.5	61.5	42.9	57.1	16.7	83.3	33.3	66.7
		Dietitian	16.7	83.3	75	25	5.6	94.4	100	0	100	0	9.1	90.9	0	100	0	100	75	25	33.3	66.7
		Pharmacist	0	100	0	100	16.7	83.3	0	100	-	-	0	100	0	100	0	100	0	100	0	100
		Midwife	-	-	-	-	-	-	-	-	100	0	-	-	-	-	-	-	-	-	-	-
8	Integrated information and education form filling		100	0	100	0	100	0	100	0	100	0	100	0	100	0	100	0	100	0	100	0
9	Medical Therapy:	Physician	100	0	75	25	83.3	16.7	100	0	50	50	54.5	45.5	0	100	100	0	100	0	100	0
	- Injection		100	0	100	0	100	0	100	0	0	100	0	100	0	100	100	0	100	0	100	0
	- Intravenous fluids		33.3	66.7	25	75	50	50	50	50	100	0	36.4	63.6	0	100	100	0	50	50	0	100
	- Oral Medication		-	-	-	-	-	-	-	-	-	-	-	-	-	-	-	-	-	-	100	0
	- Additional medication		100	0	75	25	100	0	100	0	100	0	100	0	100	0	100	0	75	25	100	0
10	Management/ Intervention	Physician	94.4	5.6	50	50	100	0	33.3	66.7	50	50	97	3	66.7	33.3	50	50	50	50	75	25
		Nurse	25	75	13.3	86.7	16.7	83.3	20	80	-	-	23.6	76.4	28.9	71.1	12.5	87.5	17.5	82.5	46.2	53.8
		Dietitian	16.7	83.3	37.5	62.5	4.2	95.8	0	100	0	100	52.3	47.7	0	100	100	0	6.3	93.7	75	25
		Pharmacist	16.7	83.3	0	100	100	0	0	100	-	-	0	100	0	100	0	100	0	100	0	100
		Midwife	-	-	-	-	-	-	-	-	100	0	-	-	-	-	-	-	-	-	-	-
11	Monitoring & Evaluation	Physician	54.2	45.8	66.7	33.3	38.9	61.1	100	0	100	0	56.8	43.2	100	0	33.3	66.7	100	0	71.4	28.6
		Nurse	100	0	100	0	100	0	100	0	-	-	100	0	100	0	100	0	100	0	100	0
		Dietitian	33.3	66.7	62.5	37.5	0	100	0	100	33.3	66.7	31.8	68.2	0	100	75	25	31.3	68.7	75	25
		Pharmacist	5.6	94.4	0	100	16.7	83.3	25	75	-	-	0	100	0	100	33.3	66.7	0	100	0	100
		Midwife	-	-	-	-	-	-	-	-	100	0	-	-	-	-	-	-	-	-	-	-
12	Mobilization/ Rehabilitation	Physician	66.7	33.3	0	100	33.3	66.7	0	100	100	0	0	100	0	100	0	100	0	100	0	100
		Nurse	83.3	16.7	37.5	62.5	33.3	66.7	0	100	-	-	0	100	33.3	66.7	0	100	0	100	50	50
		Physiotherapist	-	-	0	100	-	-	-	-	-	-	-	-	0	100	-	-	-	-	-	-
13	Outcome	Physician	58.3	41.7	50	50	72.2	27.8	100	0	100	0	59.1	40.9	75	25	33.3	66.7	100	0	66.7	33.3
		Nurse	24.1	75.9	14.6	85.4	21.4	78.6	22.2	77.8	-	-	21.2	78.8	30.6	69.4	14.3	85.7	19.4	80.6	22.2	77.8
		Dietitian	50	50	0	100	0	100	0	100	0	100	0	100	0	100	100	0	0	100	0	100
		Pharmacist	0	100	0	100	16.7	83.3	50	50	-	-	0	100	0	100	0	100	0	100	0	100
		Midwife	-	-	-	-	-	-	-	-	100	0	-	-	-	-	-	-	-	-	-	-
14	Discharge Criteria	Physician	50	50	50	50	72.2	27.8	100	0	100	0	52.3	47.7	66.7	33.3	66.7	33.3	100	0	60	40
15	Pre-Discharge Education	Nurse	44.4	55.6	25	75	100	0	83.3	16.7	-	-	100	0	55.6	44.4	0	100	75	25	100	0
		Midwife	-	-	-	-	-	-	-	-	37.5	62.5	-	-	-	-	-	-	-	-	-	-
		***Overall***	**52.2**	**47.8**	**43.1**	**56.9**	**49.0**	**51.0**	**55.5**	**44.5**	**75.2**	**24.8**	**44.3**	**55.7**	**35.6**	**64.4**	**46.8**	**53.2**	**47.8**	**52.2**	**47.8**	**52.2**
		***Mean Overall***	**55.02**										**44.46**									

*Note: n* = Frequency; *%*= Percentage; ICP = Integrated Clinical Pathway; C = Compliant; NC = Non-Compliant; PCP = Professional Care Providers; TB = Tuberculosis; COPD = Chronic Obstructive Pulmonary Disease

Labuang Baji Hospital showed lower overall compliance at 44.46%, classified as low compliance. The highest compliance rates were observed in typhoid fever and COPD diagnoses (both at 47.8%), whereas pneumonia again demonstrated the lowest compliance at 35.6%.

Qualitative interviews with PCPs revealed three primary barriers to ICP completion: (1) the perceived increase in professional responsibility and workload associated with ICP documentation, (2) insufficient staffing of essential healthcare workers, particularly clinical pharmacists in treatment areas, and (3) inadequate training and orientation regarding proper ICP completion methods across all PCPs.

### Clinical quality outcomes

Clinical quality indicators demonstrated variable compliance with ICP standards across diagnoses (Table 3). For dyspepsia, Haji Hospital patients achieved pain resolution (50%) and clinical stability (66.7%) by day 2, while the single Labuang Baji patient required 4 days. Laboratory monitoring was inadequately documented (66.7% unchecked at Haji Hospital).

**Table 3:** Clinical quality at Haji hospital and Labuang Baji hospital (*N* = 40)

No	Indicator (Outcome)	Clinical Quality							
		Haji Hospital (*n* = 20)				Labuang Baji Hospital (*n* = 20)			
		Diagnosis of the disease	*n* (%)	Days	ICP Compliant	Diagnosis of the disease	*n* (%)	Day	ICP Compliant
1	**Day 1–5**:	Dyspepsia (*n* = 6)				Dyspepsia (*n* = 1)			
	No pain		3 (50)	2	Compliant		1 (100)	4	Compliant
			2 (33.3)	3	Compliant				
			1 (16.7)	4	Compliant				
	Stable and improved condition		4 (66.7)	2	Compliant		1 (100)	4	Compliant
			1 (16.7)	3	Compliant				
			1 (16.7)	5	Compliant				
	Normal lab results		2 (33.3)	3	Compliant		1 (100)	-	**No check listed by PCP**
			4 (66.7)	-	**No check listed by PCP**				
2	**Day 1**:	Typhoid fever (*n* = 2)				Typhoid fever (*n* = 4)			
	Diagnose determined based on anamnesis and physical assessment		2 (100)	1	Compliant		4 (100)	1	Compliant
	**Day 2–5**:								
	A definitive diagnosis of salmonella typhi or paratyphi was obtained from the supporting examination		2 (100)	2	Compliant		3 (75)	2	Compliant
							1 (25)	3	Compliant
3	**Day 1–7**:	Pulmonary TB (*n* = 6)				Pulmonary TB (*n* = 11)			
	Reduced complaints		3 (50)	2	Compliant		2 (18.2)	4	Compliant
			1 (16.7)	3	Compliant		2 (18.2)	5	Compliant
			1 (16.7)	4	Compliant		2 (18.2)	6	Compliant
			1 (16.7)	5	Compliant		2 (18.2)	7	Compliant
							3 (27.3)	10	Not Compliant (Prolonged LOS)
	Improved appetite		1 (16.7)	2	Compliant		1 (9.1)	7	Not Compliant (Prolonged LOS)
							1 (9.1)	8	Not Compliant (Prolonged LOS)
			5 (83.3)	-	**No check listed by PCP**		1 (9.1)	10	Not Compliant (Prolonged LOS)
							8 (72.7)	-	**No check listed by PCP**
4	**Day 1–7**:	Pneumonia (*n* = 4)				Pneumonia (*n* = 3)			
	Reduced complaints		2 (50)	2	Compliant		2 (66.7)	3	Compliant
			2 (50)	4	Compliant		1 (33.3)	6	Compliant
	Improved lab results: Normal number of leukocytes		4 (100)	-	**No check listed by PCP**		1 (33.3)	6	Compliant
							2 (66.7)	-	**No check listed by PCP**
	Improved chest x-ray results		4 (100)	-	**No check listed by PCP**		1 (33.3)	3	Compliant
							1 (33.3)	6	Compliant
							1 (33.3)	-	**No check listed by PCP**
	No fever within 48 h, axilla temperature < 37.5ºC		3 (75)	1	Compliant		2 (66.7)	3	Compliant
			1 (25)	2	Compliant		1 (33.3)	6	Compliant
5	**Day 1–7**:	Normal Delivery (*n* = 2)				Normal Delivery (*n* = 0)			
	Normal vital signs		2 (100)	1	Compliant		-	-	-
	Controlled bleeding		2 (100)	1	Compliant		-	-	-
	No postpartum urinary retention		2 (100)	1	Compliant		-	-	-
	Good contractions		2 (100)	1	Compliant		-	-	-
6	**Day 1–7**:	COPD (*n* = 0)				COPD (*n* = 1)			
	Reduced dyspnea		-	-	-		1 (100)	8	Not Compliant (Prolonged LOS)
	Reduced cough		-	-	-		1 (100)	9	Not Compliant (Prolonged LOS)
	Improved appetite		-	-	-		1 (100)	-	**No check listed by PCP**
	Independent activity daily life		-	-	-		1 (100)	-	**No check listed by PCP**
	Reduced wheezing		-	-	-		1 (100)	8	Not compliant (Prolonged LOS)
	Normal vital signs		-	-	-		1 (100)	1	Compliant

*Note: n* = Frequency; *%*= Percentage; PCP = Professional Care Providers; ICP = Integrated Clinical Pathway; TB = Tuberculosis; COPD = Chronic Obstructive Pulmonary Disease; Systematic gaps in monitoring documentation identified across both facilities

Typhoid fever patients showed optimal performance, with diagnostic confirmation achieved on day 1–2 at both facilities (100% and 75% respectively for definitive Salmonella identification). Pulmonary TB outcomes varied significantly: Haji Hospital patients experienced symptom reduction by day 2 (50%), while Labuang Baji patients required 10 days (27.3%) due to diabetes mellitus and hypertension comorbidities. Appetite improvement monitoring was poorly documented at both sites (83.3% and 72.7% unchecked).

Pneumonia patients achieved symptom reduction within 2–3 days and fever resolution within 48 h at both hospitals (50–75% compliance). However, laboratory and radiographic follow-up documentation was consistently inadequate (100% unchecked). Normal delivery patients at Haji Hospital met all indicators by day 1 (100%). The single COPD patient at Labuang Baji required extended symptom resolution (days 8–9) due to clinical deterioration, with incomplete functional assessment documentation.

Overall, clinical outcomes aligned with ICP timeframes except when complicated by comorbidities, though systematic gaps in monitoring documentation were identified across both facilities.

### Length of stay analysis

Patient LOS outcomes demonstrated high compliance with ICP standards across both facilities (Table 4). At Haji Hospital, one dyspepsia patient (50%, class 1) exceeded ICP standards with an 8-day stay due to persistent symptoms and poor general condition. One pulmonary TB patient (16.7%) required 9 days including 2-day HCU transfer for clinical deterioration.

**Table 4:** Patient length of stays at Haji hospital and Labuang Baji hospital (*N* = 40)

No	Diagnosis	ICP-based LOS (Days)	Length of Stay (LOS)									
			Haji Hospital (*n* = 20)					Labuang Baji Hospital (*n* = 20)				
			Ward Class	*n* (%)	Day	ICP Compliant	M ± SD	Ward Class	*n* (%)	Day	ICP Compliant	M ± SD
1	Dyspepsia (*n* = 7)	1–5	Class 1 (*n* = 2)	1 (50)	2	Compliant	5.00 ± 4.24	Class 1 (*n* = 1)	1 (100)	3	Compliant	3.00
				1 (50)	8	Not Compliant						
			Class 3 (*n* = 4)	1 (25)	2	Compliant	3.50 ± 1.29	-	-	-	-	-
				1 (25)	3	Compliant						
				1 (25)	4	Compliant						
				1 (25)	5	Compliant						
2	Typhoid fever (*n* = 6)	1–6	Class 3 (*n* = 2)	1 (50)	3	Compliant	4.00 ± 1.41	Class 1 (*n* = 1)	1 (100)	5	Compliant	5.00
								Class 2 (*n* = 1)	1 (100)	6	Compliant	6.0
				1 (50)	5	Compliant		Class 3 (*n* = 2)	1 (50)	4	Compliant	5.00 ± 1.41
									1 (50)	6	Compliant	
3	Pulmonary TB (*n* = 17)	1–7	Class 3 (*n* = 6)	1 (16.7)	3	Compliant	5.33 ± 2.25	Class 1 (*n* = 1)	1 (100)	9	Not Compliant (DM & Hypertension)	9.00
				2 (33.3)	4	Compliant		Class 2 (*n* = 1)	1 (100)	11	Not Compliant	11.00
								Class 3 (*n* = 9)	2 (22.2)	3	Compliant	6.11 ± 3.33
				1 (16.7)	5	Compliant			2 (22.2)	4	Compliant	
				1 (16.7)	7	Compliant			2 (22.2)	5	Compliant	
									1 (11.1)	9	Not Compliant	
				1 (16.7)	9	Not Compliant			1 (11.1)	10	Not Compliant	
									1 (11.1)	12	Not Compliant	
4	Pneumonia (*n* = 7)	1–7	Class 3 (*n* = 4)	1 (25)	3	Compliant	4.25 ± 0.957	Class 2 (*n* = 1)	1 (100)	5	Compliant	5.00
				1 (25)	4	Compliant		Class 3 (*n* = 2)	1 (50)	4	Compliant	5.50 ± 2.12
				2 (50)	5	Compliant			1 (50)	7	Compliant	
5	Normal Delivery (*n* = 2)	1–7	Class 2 (*n* = 1)	1 (100)	1	Compliant	1.00	-	-	-	-	-
			Class 3 (*n* = 1)	1 (100)	1	Compliant	1.00	-	-	-	-	-
6	COPD (*n* = 1)	1–7	-	-	-	-	-	Class 2 (*n* = 1)	1(100)	9	Not compliant	9.00

*Note: n* = Frequency; %= Percentage; *M* = Mean; *SD* = Standard Deviation; ICP = Integrated Clinical Pathway; TB = Tuberculosis; COPD = Chronic Obstructive Pulmonary Disease; Extended stays consistently associated with patient comorbidities, clinical complications, or poor baseline conditions rather than care delivery factors

Labuang Baji Hospital showed greater variability in pulmonary TB cases. Class 1 patients experienced extended stays (9 days) attributed to comorbid diabetes mellitus and hypertension. Class 2 and 3 patients required 11 and 12 days respectively due to prolonged symptom duration and poor general condition. One COPD patient exceeded standards (9 days) due to malnutrition (low BMI) and clinical instability.

All typhoid fever cases (3–6 days), pneumonia cases, and normal delivery cases across both hospitals maintained ICP-compliant LOS ranges. Extended stays were consistently associated with patient comorbidities, clinical complications, or poor baseline conditions rather than care delivery factors.

### Hospital cost analysis

Table 5 shows hospital costs at Haji Hospital varied across diagnoses and patient care classes. The implementation of ICP resulted in cost-effectiveness, with hospitals achieving profit differences in all disease diagnoses across care classes. The highest cost was for Pulmonary TB patients in class 3 care at IDR 4,584,800 (approx. USD 276.6), while the lowest was for dyspepsia patients in class 3 care at IDR 529,000 (approx. USD 31.9).

**Table 5:** Hospital costs at Haji hospital (*n* = 20)

No	Diagnosis	Ward Class	INA-CBG Tariff (IDR)	Hospital Cost		
				Hospital Expenses (IDR)	Differences in INA-CBG Tariff and Hospital Expenses (IDR)	INA-CBG Compliant
1.	Dyspepsia (*n* = 6)	Class 1 (*n* = 2)	1,998,400	844,000	1,154,400	Compliant
				2,899,000	**− 900**,**600**	Not compliant (Prolonged LOS up to 8 days)
		Class 3 (*n* = 4)	1,427,400	**529**,**000**	898,400	Compliant
				970,500	456,900	Compliant
				1,274,000	153,400	Compliant
				1,450,000	**-22**,**600**	Not compliant
2.	Typhoid fever (*n* = 2)	Class 3 (*n* = 2)	2,229,100	790,000	1,439,100	Compliant
				1,352,500	876,600	Compliant
3.	Pulmonary TB (*n* = 6)	Class 3 (*n* = 6)	4,260,700	1,730,000	2,530,700	Compliant
				1,413,000	2,847,700	Compliant
				2,622,000	1,638,700	Compliant
				2,207,000	2,053,700	Compliant
				3,302,000	958,700	Compliant
				**4**,**584**,**800**	**-323**,**100**	Not compliant (Prolonged LOS up to 9 days)
4.	Pneumonia (*n* = 4)	Class 3 (*n* = 4)	3,550,100	1,232,000	2,318,100	Compliant
				1,298,000	2,252,100	Compliant
				1,788,000	1,762,100	Compliant
				1,813,000	1,737,100	Compliant
5.	Normal Delivery (*n* = 2)	Class 2 (*n* = 1)	2,753,300	1,539,000	1,214,300	Compliant
		Class 3 (*n* = 1)	2,363,300	1,308,000	1,055,300	Compliant

*Note: n* = Frequency; IDR = Indonesian Rupiah; INA-CBGs = Indonesian Case-Based Groups; TB = Tuberculosis; ICP implementation resulted in cost-effectiveness with profit differences in most disease diagnoses; Highest cost: Pulmonary TB class 3 care (IDR 4,584,800 ≈ USD 276.6); Lowest cost: Dyspepsia class 3 care (IDR 529,000 ≈ USD 31.9)

ICP implementation at Labuang Baji Hospital demonstrated overall financial benefits despite variable individual case outcomes (Table 6). The highest treatment cost occurred in COPD patients (class 2 care) at IDR 6,903,838 (USD 416.6), exceeding Indonesian Case-Based Groups (INA-CBGs) rates by IDR 2,554,638 (USD 154.2) due to extended 9-day LOS. Conversely, dyspepsia patients (class 1 care) generated the lowest costs at IDR 1,577,273 (USD 95.2), yielding profit margins of IDR 421,127 (USD 25.4).

**Table 6:** Hospital cost at Labuang Baji hospital (*n* = 20)

No	Diagnosis	Ward Class	INA-CBG Tariff (IDR)	Hospital Cost		
				Hospital Expenses (IDR)	Differences in INA-CBG Tariff and Hospital Expenses (IDR)	INA-CBG Compliant
1.	Dyspepsia (*n* = 1)	Class 1 (*n* = 1)	1,998,400	**1**,**577**,**273**	421,127	Compliant
		Class 2 (*n* = 0)	1,712,900	-	-	-
		Class 3 (*n* = 0)	1,427,400	-	-	-
2.	Typhoid fever (*n* = 4)	Class 1 (*n* = 1)	3,120,700	2,453,520	667,180	Compliant
		Class 2 (*n* = 1)	2,674,900	2,880,242	**-205**,**342**	Not compliant
		Class 3 (*n* = 2)	2,229,100	2,330,314	**-101**,**214**	Not compliant
				1,810,942	418,158	Compliant
3.	Pulmonary TB (*n* = 11)	Class 1 (*n* = 1)	5,965,000	6,259,033	**-294**,**033**	Not compliant (prolonged LOS up to 9 days)
		Class 2 (*n* = 1)	5,112,900	4,196,995	915,905	Compliant
		Class 3 (*n* = 9)	4,260,700	1,679,597	2,581,103	Compliant
				2,019,687	2,241,013	Compliant
				2,047,303	2,213,397	Compliant
				2,819,596	1,441,104	Compliant
				1,934,896	2,325,804	Compliant
				3,689,566	571,134	Compliant
				4,347,342	**-86**,**642**	Not compliant (prolonged LOS up to 9 days)
				2,635,377	1,625,323	Compliant
				3,892,473	368,227	Compliant
4.	Pneumonia (*n* = 3)	Class 2 (*n* = 1)	4,260,100	2,645,207	1,614,893	Compliant
		Class 3 (*n* = 2)	3,550,100	2,719,558	830,542	Compliant
				4,020,200	**-470**,**100**	Not compliant
5.	COPD (*n* = 1)	Class 2 (*n* = 1)	4,349,200	**6**,**903**,**838**	**-2**,**554**,**638**	Not compliant (prolonged LOS up to 9 days)

*Note: n* = Frequency; IDR = Indonesian Rupiah; INA-CBGs = Indonesian Case-Based Groups; TB = Tuberculosis; COPD = Chronic Obstructive Pulmonary Disease; Overall financial benefits despite variable individual case outcomes; Net profitability across most diagnostic categories; Only Pulmonary TB in class 1 care producing financial losses; Highest cost: COPD class 2 care (IDR 6,903,838 ≈ USD 416.6); Lowest cost: Dyspepsia class 1 care (IDR 1,577,273 ≈ USD 95.2)

Aggregate analysis revealed net profitability across most diagnostic categories and care classes, with only pulmonary TB in class 1 care producing financial losses. These findings indicate that ICP implementation provides sustainable economic benefits for the hospital, with cost overruns in complex cases offset by efficiency gains in routine diagnoses.

### Patient satisfaction

**Fig. 1:**
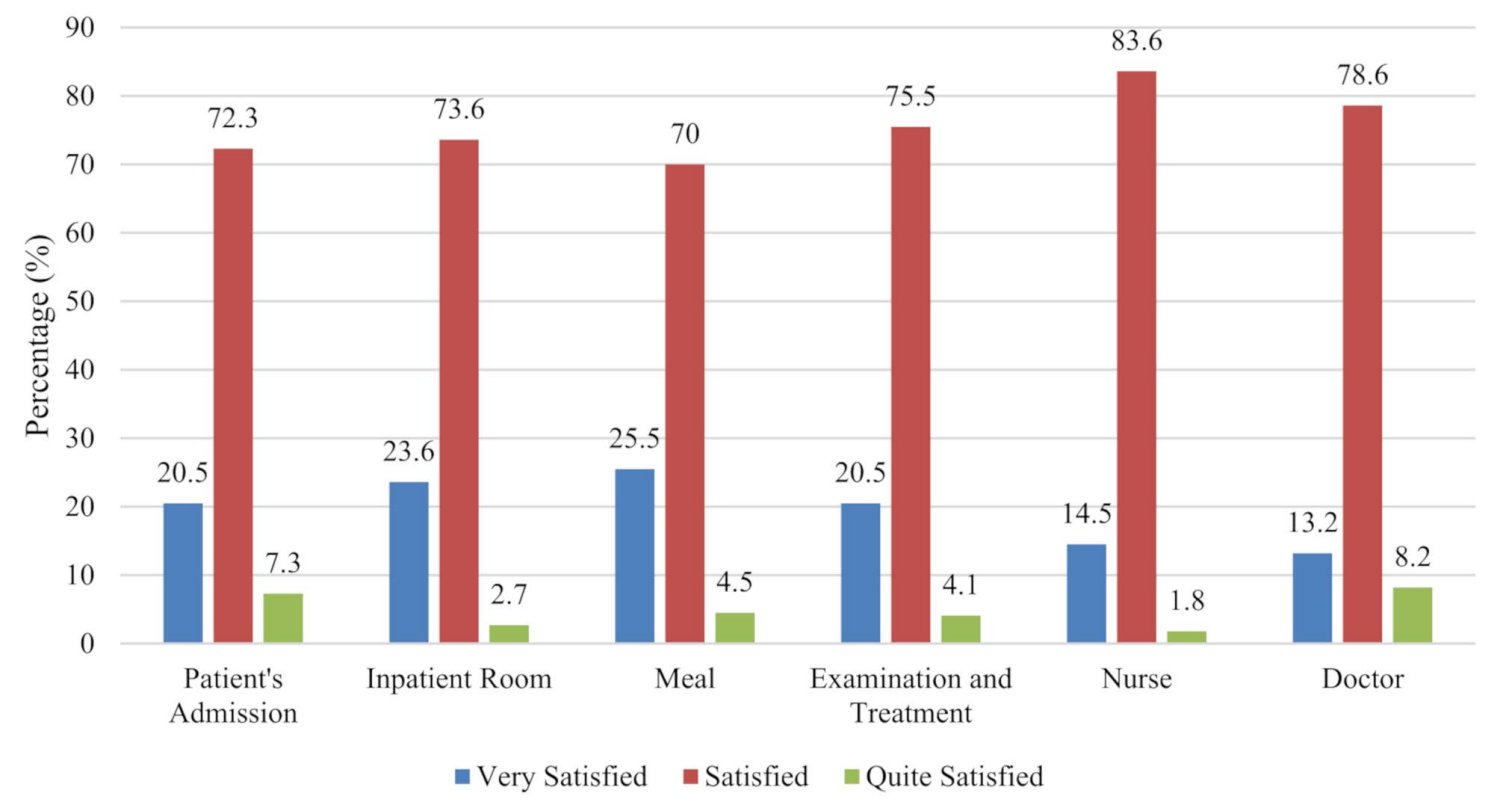
Patient satisfaction at Haji hospital (*n* = 20). *Note:* Categories defined based on predefined score ranges using validated questionnaire adapted from previous study with *Cronbach’s alpha* (*α* = 0.767)

**Fig. 2:**
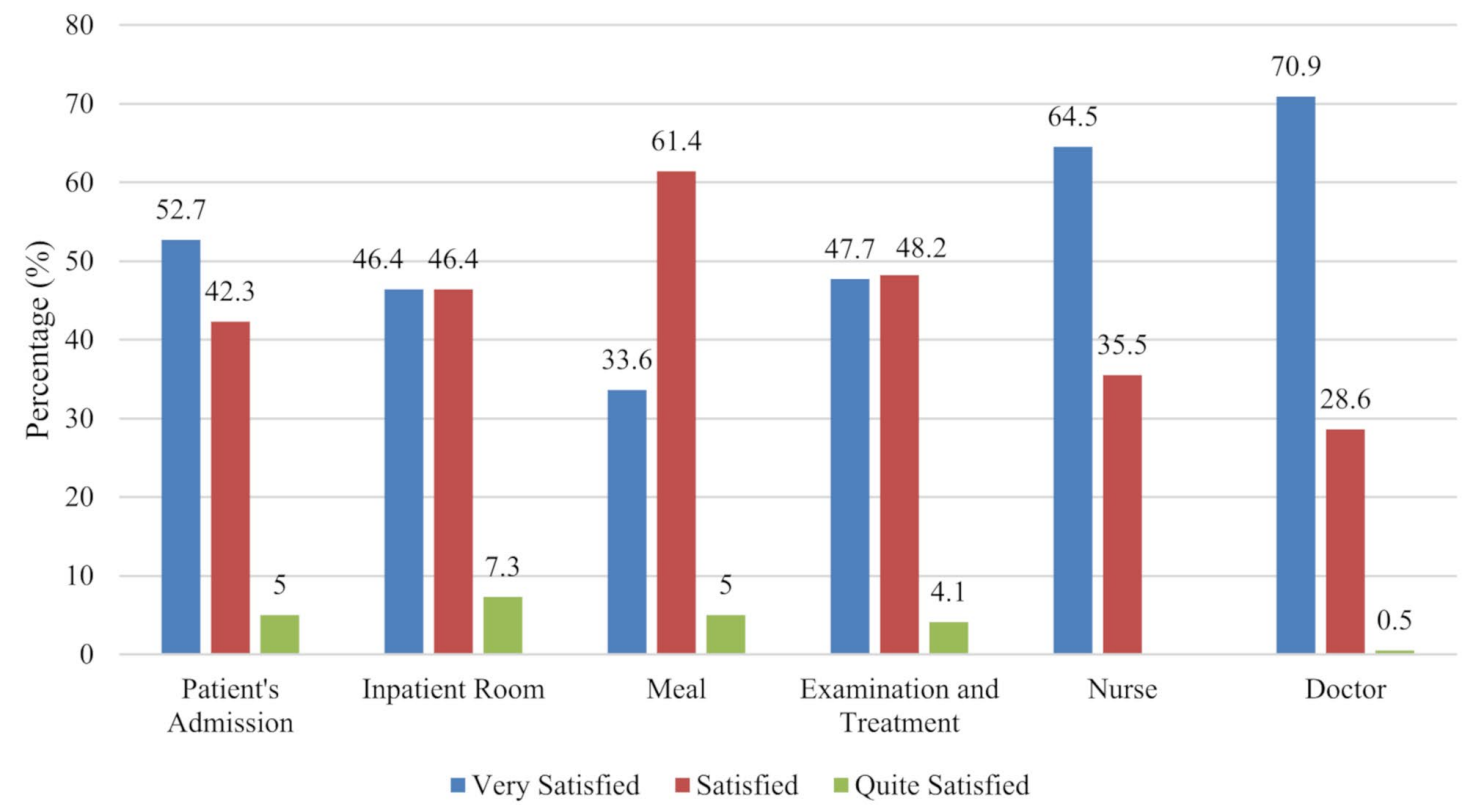
Patient Satisfaction at Labuang Baji Hospital (*n* = 20). *Note:* Categories defined based on predefined score ranges using validated questionnaire adapted from previous study with *Cronbach’s alpha* (*α* = 0.767)

Figures 1 and 2 show that most patients were satisfied with services after ICP implementation. At Haji Hospital, health workers (nurses) achieved the highest satisfaction percentage at 83.6%. At Labuang Baji Hospital, patients felt very satisfied with physicians (70.9%), though some patients felt only quite satisfied with inpatient room facilities (7.3%) due to concerns about facility quality and cleanliness.

## Discussion

### Main findings

This study demonstrates that ICP implementation at both hospitals resulted in improved clinical outcomes, generally appropriate length of stay, cost-effectiveness in most diagnoses, and enhanced patient satisfaction, despite suboptimal compliance rates with ICP protocols.

### Clinical outcomes and length of stay

The implementation of ICPs led to improved patient clinical outcomes, with many patients achieving recovery milestones within specified timeframes or even earlier than ICP standards. In this study, it was found that there were some patients who went home earlier than the standard ICP treatment day because the patient’s clinical outcomes improved after a few days of receiving treatment. This is possible because the ICP provides an effective standard of care and drug use from collaboration between care professionals. After the implementation of the clinical pathway, it was found that the cost of hospitalization, surgery, treatment, and medical consumables even patients had a shorter length of hospital stay and a significant reduction in the duration of antibiotic use, thus positively impacting patient clinical outcomes [20, 21]. Research in China found the introduction of clinical pathways can shorten patient care days by an average difference of two days [22]. In hospitalized patients with a diagnosis of Community Acquired Pneumonia (CAP), the average LOS in the pre-clinical pathway period is 5 days (range 3–16) and in the post-clinical pathway period of 4 days (range 2–14) [23]. It was also found in studies in patients with post pancreatectomy, pulmonary embolism, and acute myocardial infarction that showed a decrease in the duration of hospitalization after the implementation of the clinical pathway [24–26]. In addition, the implementation of the clinical pathway has the potential to reduce complications, readmission within 30 days after the patient is discharged, and length of stay without affecting mortality or quality of life after the implementation of the clinical pathway compared to the usual care group [27–29].

Some patients experienced extended LOS beyond ICP standards due to underlying conditions, comorbidities such as diabetes mellitus and hypertension, and slower recovery processes. This is consistent with research indicating that higher disease severity may reduce the influence of clinical pathway implementation on length of stay [30].

### Cost-effectiveness

ICP implementation positively impacted hospital financial outcomes, with most diagnoses generating profit differences when compared to INA-CBGs rates. The reduction in LOS directly influenced accommodation costs, leading to overall cost savings. This finding supports previous research on clinical pathway implementation in asthma patients, which showed shorter hospitalization duration and lower hospital costs [31]. Studies on caesarean section patients with complete versus incomplete clinical pathway implementation also demonstrated that complete pathways resulted in shorter average hospitalization and significantly lower costs [32]. Although hospitalization days decreased, there was no increase in morbidity, rehospitalization or death. In addition, hospitalization costs decreased and helped effectively reduce medical resource burdens and national insurance pressures and the average hospitalization index decreased by 22% [22, 24].

### Patient satisfaction

Patient satisfaction improved across multiple domains following ICP implementation. Factors contributing to satisfaction included prompt emergency room services, attentive healthcare workers, consistent care delivery, and clear examination and treatment services. This improvement reflects enhanced trust in physicians and nurses, as well as improved communication between healthcare providers [33]. Effective physician-patient communication and reasonable medical costs contribute to higher satisfaction and increased likelihood of hospital recommendations [34].

### ICP Implementation challenges and strategic recommendations

Low compliance rates (55.02% at Haji Hospital and 44.46% at Labuang Baji Hospital) represent a significant implementation challenge. The identified barriers include increased perceived responsibility, insufficient staffing (particularly clinical pharmacists), and inadequate training on ICP completion methods. These findings emphasize the importance of comprehensive implementation strategies, including quality analysis, additional training, and improved communication to strengthen multidisciplinary team collaboration [35].

The implementation of clinical care pathways for stages in screening, diagnosis, and treatment has been shown to improve the quality of health care delivery in medicine, making it important to address treatment processes that are often inconsistent [36]. In addition, the implementation of clinical pathways can decrease annual hospitalization time per patient possibly due to better patient discharge planning.

 [30]. Effective implementation of the clinical pathway requires compliance from nursing care professionals. Adherence to care has been shown to improve clinical outcomes, internal and external support from hospitals is needed to facilitate the implementation of clinical pathways for inpatients [37].

### Strategic framework for enhanced implementation

Based on these findings and contemporary evidence, we propose a comprehensive six-component implementation strategy informed by current best practices. Communication skills training has been identified as an effective strategy to improve care provided by professionals in patient care and the quality of health services, with systematic evidence demonstrating that training programs can promote changes in attitude, behavior, and self-efficacy among health professionals [38]. Recent intervention studies demonstrate that multidisciplinary team training focusing on communication and creating psychologically safe environments can facilitate challenging communication scenarios, prevent patient safety risks, and increase team performance perception [39].

The framework encompasses: comprehensive staff training on ICP benefits and methodologies; adequate clinical pharmacist staffing; simplified documentation processes; regular compliance monitoring with feedback systems; enhanced interdisciplinary communication protocols; and recognition programs for high-performing teams. Contemporary evidence emphasizes that healthcare professionals who are already qualified need development of specific skills to work effectively as part of diverse teams and to identify and overcome barriers to effective teamwork [40]. Successful implementation requires standardized dissemination templates and customized plans specific to pathway content and scope, paired with key stakeholder identification across departments and units [41].

### Study strengths and limitations

This investigation provides valuable real-world evidence through comprehensive evaluation across multiple outcome domains in two distinct hospital settings, utilizing validated data collection instruments. The hospital cost analysis demonstrated preliminary financial benefits with overall cost savings despite variable individual case outcomes.

However, several limitations warrant consideration. The small sample size and single country setting limit generalizability to healthcare systems with different organizational structures and payment mechanisms. The observational design precludes causal inference, while the short follow-up period may not capture long-term sustainability or delayed cost impacts. Recent systematic reviews emphasize that reduced length of stay appears associated with clinical pathways, though evidence remains unclear regarding quality-of-life improvements and patient satisfaction across different conditions [42]. The hospital cost analysis was limited to direct treatment costs and did not encompass broader economic perspectives including indirect costs, productivity impacts, or societal costs.

### Future research directions

Future investigations should prioritize methodologically robust designs to advance implementation of science and cost analysis methodologies. Systematic evidence indicates that clinical pathway software implementation primarily increases adherence, which may positively impact length of stay and diagnostic effectiveness, though effects on costs and patient outcomes remain inconclusive requiring further research [43]. Large-scale multicenter randomized controlled trials incorporating diverse healthcare settings and payment systems are essential to establish causal relationships.

Hospital cost analyses should concentrate on comprehensive costs of development and implementation, as clinical pathway development consumes considerable resources when done as recommended in active processes but likely produces positive effects on patient outcomes while reducing hospital costs [44]. Evidence demonstrates that pathway care costs increased approximately 1% annually compared to 6–7% for non-pathway care, indicating substantial cost control potential [45]. Future studies should expand beyond hospital cost analysis to include comprehensive economic evaluations incorporating productivity losses, quality-adjusted outcomes, and long-term healthcare utilization.

Priority research questions include optimal implementation strategies achieving sustainable compliance rates above 80%, standardized metrics for measuring cost-effectiveness ratios, and comparative cost analysis examining different pathway designs across healthcare contexts to inform evidence-based resource allocation decisions.

## Conclusions

The implementation of ICP in multiple diagnoses at Haji Hospital and Labuang Baji Hospital demonstrated positive impacts on patient clinical outcomes, length of stay, hospital costs, and patient satisfaction. While compliance with ICP protocols remained suboptimal, the intervention showed promise for improving healthcare quality and efficiency. Key recommendations for future implementation include enhanced staff training, adequate staffing, simplified documentation processes, and regular compliance monitoring to maximize ICP benefits.

To improve ICP implementation success, hospitals should focus on comprehensive training programs, adequate resource allocation, streamlined documentation systems, and robust quality monitoring mechanisms. These strategies will help overcome identified barriers and enhance the effectiveness of ICP-based care delivery models.

## Data Availability

The datasets used and/or analyzed during the current study are available from the corresponding author on reasonable request.

## Abbreviations

CDs: Communicable Diseases
COPD: Chronic Obstructive Pulmonary Disease
DM: Diabetes Mellitus
ICP: Integrated Clinical Pathways
INA-CBGs: Indonesian Case-Based Groups
LOS: Length of Stay
NCDs: Non-Communicable Diseases
NHI: National Health Insurance
PCP: Professional Care Providers
TB: Tuberculosis
